# Cardiometabolic sex differences in adults born small for gestational age

**DOI:** 10.3389/fcvm.2023.1223928

**Published:** 2023-10-25

**Authors:** Mérida Rodríguez-López, Álvaro Sepúlveda-Martínez, Gabriel Bernardino, Francesca Crovetto, Carolina Pajuelo, Marta Sitges, Bart Bijnens, Eduard Gratacós, Fàtima Crispi

**Affiliations:** ^1^ BCNatal - Barcelona Center for Maternal-Fetal and Neonatal Medicine (Hospital Clínic and Hospital Sant Joan de Deu), Centro de Investigaciones Biomédica en Red – Enfermedades Raras, Universitat de Barcelona, Barcelona, Spain; ^2^ Faculty of Health Science, Universidad Icesi, Cali, Colombia; ^3^ Clinical Research Center, Fundación Valle del Lili, Cali, Colombia; ^4^ Fetal Medicine Unit, Department of Obstetrics and Gynecology, Hospital Clínico Universidad de Chile, Santiago de Chile, Chile; ^5^ BCN Medtech, Department of Information and Communication Technologies, Universitat Pompeu Fabra, Barcelona, Spain; ^6^ Institut Clínic Cardiovascular, Hospital Clínic, Centre for Biomedical Research on CardioVascular Diseases (CIBERCV), Universitat de Barcelona, Barcelona, Spain; ^7^ Institut d’Investigacions Biomèdiques August Pi I Sunyer, Barcelona, Spain; ^8^ Catalan Institution for Research and Advanced Studies (ICREA), Barcelona, Spain

**Keywords:** fetal programming, cardiovascular disease, sex differences, low birth weight, echocardiography, electrocardiography, blood pressure

## Abstract

**Aim:**

This study aimed to assess the cardiometabolic sex similarities and differences in adults born small for gestational age.

**Methods:**

This study was an ambispective cohort study from a birth registry in Barcelona, Spain, including 523 adult participants (20–40 years-old) subdivided as born small for gestational age (SGA, if birth weight <10th centile) or adequate fetal growth for gestational age (AGA). Cardiometabolic health was assessed by echocardiography, electrocardiogram, blood pressure measurement, vascular ultrasound, anthropometric measurements, and serum glycemia and lipid profile. Stratified analyses by sex were performed by estimation of adjusted absolute difference (AAD) using inverse probability weighting.

**Results:**

Compared with AGA, the stratified analyses by sex showed a more pronounced reduction in ejection fraction [AAD: female −1.73 (95% CI −3.2 to −0.28) vs. male −1.33 (−3.19 to 0.52)] and increment in heart rate [female 3.04 (0.29–5.8) vs. male 2.25 (−0.82 to 5.31)] in SGA females compared with SGA males. In contrast, a more pronounced reduction in PR interval [female −1.36 (−6.15 to 3.42) vs. male −6.61 (−11.67 to −1.54)] and an increase in systolic blood pressure [female 0.06 (−2.7 to 2.81) vs. male 2.71 (−0.48 to 5.9)] and central-to-peripheral fat ratio [female 0.05 (−0.03 to 0.12) vs. male 0.40 (0.17–0.62)] were mainly observed in SGA male compared with SGA female.

**Conclusions:**

Sex differences were observed in the effect of SGA on cardiometabolic endpoints with female being more prone to cardiac dysfunction and male to electrocardiographic, vascular, and metabolic changes. Future research including sex-stratification data is warranted.

## 1. Introduction

Sex differences are present in the incidence and prognosis of cardiovascular diseases (CVDs). While CVD is usually less frequent in females, female show higher rates of mortality and hospitalization and worse prognosis after acute cardiovascular events (1). Reduced patient awareness and clinician bias are potential important factors for delayed diagnosis and treatment and worse prognosis in female (2–4). Furthermore, differences in the type of cardiometabolic events are potential reasons for the sex-related differences: female are more prone to develop heart failure, while male usually have a twofold higher incidence of coronary heart disease (5, 6), arrhythmogenic cardiomyopathies, hypertension, and hypercholesterolemia compared with female (7). Common and female-specific risk factors in adulthood have been proposed to explain such differences. While women's hormones are cardioprotective, only females are prone to reproductive endocrine disorders including polycystic ovary syndrome, menopause, and pregnancy related disorders (8). Most research has focused on the postmenopausal period, when CVD usually is evident in females; however, whether sex differences can arise at earlier stages of life has been poorly studied.

Strong evidence indicates that CVD starts early in life, long before the clinical symptoms appear, and can even start before birth (9). The early origins of adult heart disease have been identified by pioneering work in the late 1980s, which established that rates of death from adult heart disease are ordered across the birth weight scale (10). The association between birthweight and cardiovascular risk in adults was denominated *fetal programming* or the early origins of adult cardiometabolic disease. An independent association between being born too small (small for gestational age, SGA) and cardiovascular remodeling has been demonstrated in fetal life (11), childhood (12), and more recently in young adulthood (13). However, whether male and female differ in the programmed cardiovascular risk is not clearly understood and there are contradictory findings. Similar inverse associations of birthweight with blood pressure (14) and CVD (15) have been reported for both male and female. In contrast, a cohort study reported a stronger inverse association between birthweight and coronary heart disease and stroke in female (16). Moreover, worse coronary heart disease risk score (17) and lower cardiorespiratory fitness (18) have been reported in young male born with low birthweight compared with female. Animal models suggest that female offspring might be protected from the adverse effects of fetal insult (19), and that male are more prone to hypertension (20). However, signs of left ventricular diastolic dysfunction and pulmonary hypertension has been also reported in both females and males (21). Thus, studies approaching the influence of sex on fetal cardiovascular programming related to SGA are limited and inconsistent.

Our aim was to explore the cardiometabolic sex similarities and differences in adults born SGA vs. those born with an adequate birthweight for gestational age (AGA). To achieve this aim, we conducted a sex-stratified analysis in a well-phenotype cohort of adults born SGA and AGA.

## 2. Material and methods

### 2.1. Study design and participants

An ambispective cohort study was conducted in adults born between 1975 and 1995 from a birth registry at a tertiary university hospital in Barcelona, Spain. SGA was defined as birth weight <10th centile for gestational age. Unexposed individuals with appropriate fetal growth for gestational age (AGA) were defined as birth weight ≥10th centile. Two different birthweight standards were used: local standards from 1975 to 1995 (22) (contemporary but limited as not customized by sex and with small sample sizes at extreme gestational ages), and current local standards customized by gestational age and sex (23). Participants with discordant birthweight centile results on the two standards, congenital malformations, structural or chromosomal abnormalities, twin pregnancies, professional athletes, major mental disorder, and those with birthweight >95th centile were excluded. To prevent the inclusion of constitutionally small cases, those born from mothers with a height <1.46 m were also excluded. Biological sex was obtained from the birth register. Self-reported gender coincided with birth recorded biological sex in all participants. The study was approved by the Ethical Committees of Hospital San Joan de Déu and Hospital Clinic in Barcelona, Spain.

### 2.2. Enrollment

The birth register contained birthdate, demographic, pregnancy, and perinatal information. Deliveries were recorded following birthdate order and each birth was identified by a consecutive number throughout the years. First, a random selection was performed using this identification number. Then, the birth weight percentile was determined, and subjects were classified as SGA or AGA. As this selection generated more AGA than SGA, additional SGA cases were randomly selected from among those that were born within the same year as those born AGA. Next, each child's surname was obtained from mother's clinical record. Based on birthdate and surname, the participants were located in the local register of the relevant health affiliation and contact data obtained. Thereafter, a letter of invitation was sent to the participants and they were subsequently contacted via phone calls. If the individual was interested in participating, inclusion and exclusion criteria were verified. Then, a detailed explanation of the study protocol was provided and informed consent signed.

### 2.3. Sample size

Sample size was calculated assuming an unknown but equal variance [as suggested by previous studies (12, 24)], 80% power, 5% alpha-error, 1:1 allocation index. A standardized mean difference of 0.25 was used to maximize the sample size. Then, a sample size of 250 SGA and 250 AGA individuals was estimated for echocardiography. Assuming an overall response and recruitment rate of ∼25%, 2,000 patients were initially selected. The stratified analysis was unplanned, therefore, the width and precision of the 95% confidence interval (95% CI) of measures of associations were considered for the interpretation of the data (25, 26).

### 2.4. Study protocol

The study protocol included echocardiography, electrocardiogram (ECG), measurement of blood pressure, vascular ultrasound, medical examination including a questionnaire, anthropometry, and collection of peripheral blood samples. These measurements were obtained by a trained physicians and cardiologists blinded to the individual's SGA categories. In addition, covariables were obtained via participant interviews and reviewing birth medical records.

### 2.5. Cardiovascular assessment

#### 2.5.1. Echocardiography

Each participant underwent a comprehensive echocardiography performed by an experienced cardiologist using a commercially available ultrasound scanner (Vivid E9, General Electric Healthcare) with a 2.5 MHz transducer (M5S). Standard echocardiographic views were obtained with the patient in left lateral decubitus position, and images were analyzed offline with a commercially available software (EchoPac, General Electric Healthcare. version 108.1.6) by an experienced observer unaware of exposure status. Atrial and ventricular dimensions were measured following the European Society of Cardiology guidelines (27), and 2D sphericity indices were calculated as base-to-apex length/basal diameter. Relative wall thickness was calculated as [posterior left ventricular (LV) wall thickness*2]/end-diastolic LV cavity diameter. Systolic and diastolic function were assessed by measuring ejection fraction, cardiac output, annular excursion and velocities, filling E/A ratios, and isovolumic relaxation time. Left ventricular ejection fraction was estimated by the biplane method (27). LV stroke volume and cardiac output were obtained from aortic Doppler imaging. Right ventricular (RV) fractional area change was calculated as the difference in end-diastolic area and end-systolic area divided by the end-diastolic area, from the apical four-chamber view (28). Mitral and tricuspid longitudinal annular motion were assessed by M-mode and real-time tissue Doppler from an apical four-chamber view. Peak early (E) and late (A) transvalvular filling velocities were obtained, and E/A ratio calculated. Isovolumic relaxation time was measured from the end of the aortic/pulmonary wave to the beginning of the mitral/tricuspid early filling wave. Cardiac morphometric parameters were indexed by body surface area (BSA). Dubois formula was used to calculate BSA as (weight) kg^0.425^ × (height) m^0.725^ × 0.007184.

#### 2.5.2. Electrocardiographic assessment

Digital standard 12-lead surface ECGs were recorded at rest using a Gem Heart One recorder (Gem-Med SL. Spain) at an equivalent paper speed of 50 mm/s and a gain of 10 mm/mV. Waves and interval duration were automatically measured by the ECG system.

#### 2.5.3. Blood pressure

Peripheral blood pressure was measured non-invasively (Infunix, IP1020) by a trained nurse over the right brachial artery and after 5–10 min resting. The mean of three consecutive systolic and diastolic values was used for the analysis.

#### 2.5.4. Carotid ultrasound

Left and right carotid artery cines were obtained by ultrasound at 1 cm proximal to the bifurcation of the common carotid artery (GE Vivid q). Carotid intima-media thickness (cIMT) was measured offline based on an automatic tracing method with dedicated software (EchoPAC. General Electric Healthcare. version 108.1.x). Three end-diastolic frames were selected and analyzed. The average measurement was calculated for both right and left cIMT. The mean value of both sides was used for the analysis.

### 2.6. Anthropometric and metabolic assessment

#### 2.6.1. Body anthropometry

Body mass index was calculated and further classified as overweight or obese if the value was >25 kg/m^2^ and obese if >30 kg/m^2^. An inelastic tape (Seca®, CA, USA) was used to measure the waist and hip circumferences, while the patient maintained the feet together and with the weight distributed equally on both feet. Waist circumference was measured horizontally midway between the lowest rib and the iliac crest. Hip circumference was measured on the area of greatest gluteal circumference. Waist-to-hip ratio was calculated as a surrogate of central obesity. Dubois formula was used to calculate body surface area (BSA) as (weight in kg) 0.425 × (height in m) 0.725 × 0.007184.

Skinfolds were measured with a Harpenden plicometer calibrated to 0.2 mm on the left side of the body. Brachial skinfold was measured on the mid proportion of the arm, tricipital in the middle between the acromion and olecranon, subscapular at 2 cm below the angle of the scapula, and suprailiac at 2 cm above the iliac crest and 3 cm towards the umbilicus. The central-to-peripheral fat ratio was obtained by the formula: (iliac + scapular)*100/(biceps + triceps). In addition, lean, muscular, and fat mass was measured using an electronic scale (Tanita. Body Composition Analyzer. BC-420MA. Japan). Fit-to-fit bioelectric impedance was obtained directly from the Tanita algorithm.

#### 2.6.2. Laboratory

Peripheral blood samples were obtained by venipuncture. Serum was separated by centrifugation at 2,000 g for 10 min and samples aliquots were immediately stored at −80°C until analyzed. Sera concentrations of glucose, total cholesterol, high density lipoprotein (HDL), and triglycerides were analyzed using standardized enzyme-linked immunoSorbent assay (ELISA).

### 2.7. Covariables

Perinatal data was obtained from the delivery register. Hypertensive disorder in pregnancy included preeclampsia/eclampsia or pregnancy hypertension according to the birth register. Current characteristics including sociodemographic and personal antecedents were obtained by a structured questionnaire. Family history of cardiovascular disease was self-reported and was considered as positive whether any of the participant's parents suffered from myocardial infarction, hypercholesterolemia, diabetes, or hypertension. Physical activity was assessed by the international physical activity questionnaire (short version) (29). Physical activity was classified as: high (vigorous activity on at least 3 days achieving a minimum total physical activity of at least 1,500 metabolic equivalent of task (MET)-minutes/week, or five or more days of any combination of walking or moderate or vigorous activities that achieve at least 3,000 MET-minutes/week), moderate (at least 20 min of vigorous activity per day for three or more days per week, or at least 30 min of moderate intensity activity per day for five or more days per week, or five or more days of any combination of walking, moderate-intensity, or vigorous intensity activities achieving at least 600 MET minutes/week). When the participants did not meet the above criteria, they were classified as sedentary.

### 2.8. Statistical analysis

Stata 14.0 (StataCorp. LP. College Station. TX) was used for statistical analysis. We first determined the difference between AGA and SGA in the overall population and a stratified analysis by sex was performed similarly. For quantitative variables, normality assumption was checked using the Shapiro–Wilk test. Then, study groups were described using mean (standard deviation) or median (interquartile range). Student's *t*-test or the Mann–Whitney test were used to compare the mean/median, respectively. The *X*^2^ test and the Fisher's exact test were used as appropriate for comparison of categorical variables. A *P* value <0.05 was accepted as statistically significant. Kernel-weighted local polynomial regression was used to explore the relationship between heart rate and blood pressure by categories of sex and SGA background.

For each variable, an inverse probability weighting (IPW) was used with the aim of achieving a balanced distribution of confounders across exposure groups and simulating a randomized trial. Weights were assigned to individuals based on the inverse of their probability of being SGA, using a propensity score probit model. Variables that are both related to the exposure and to the exposure–outcome relationship were included in the model. This results in a pseudo-population in which patients with a high probability of being exposed have a smaller weight and patients with a low probability of exposure have a larger weight and thus the distribution of measured patient characteristics used to calculate the propensity score becomes independent of the exposure.

The average exposure effect with robust standard errors was then estimated and called the adjusted absolute difference (AAD), i.e., the average effect of moving an entire population from AGA to SGA (30). Standardized mean differences (SMD) and the variance ratio were used to verify the baseline covariates balance between SGA and AGA in the overall and stratified analyses. As a rule of thumb, the SMD should be lower than 0.1 and the variance ratio close to 1. Overall balance was also inspected by the overidentification test (31, 32).

## 3. Results

### 3.1. Study population

**Figure 1:**
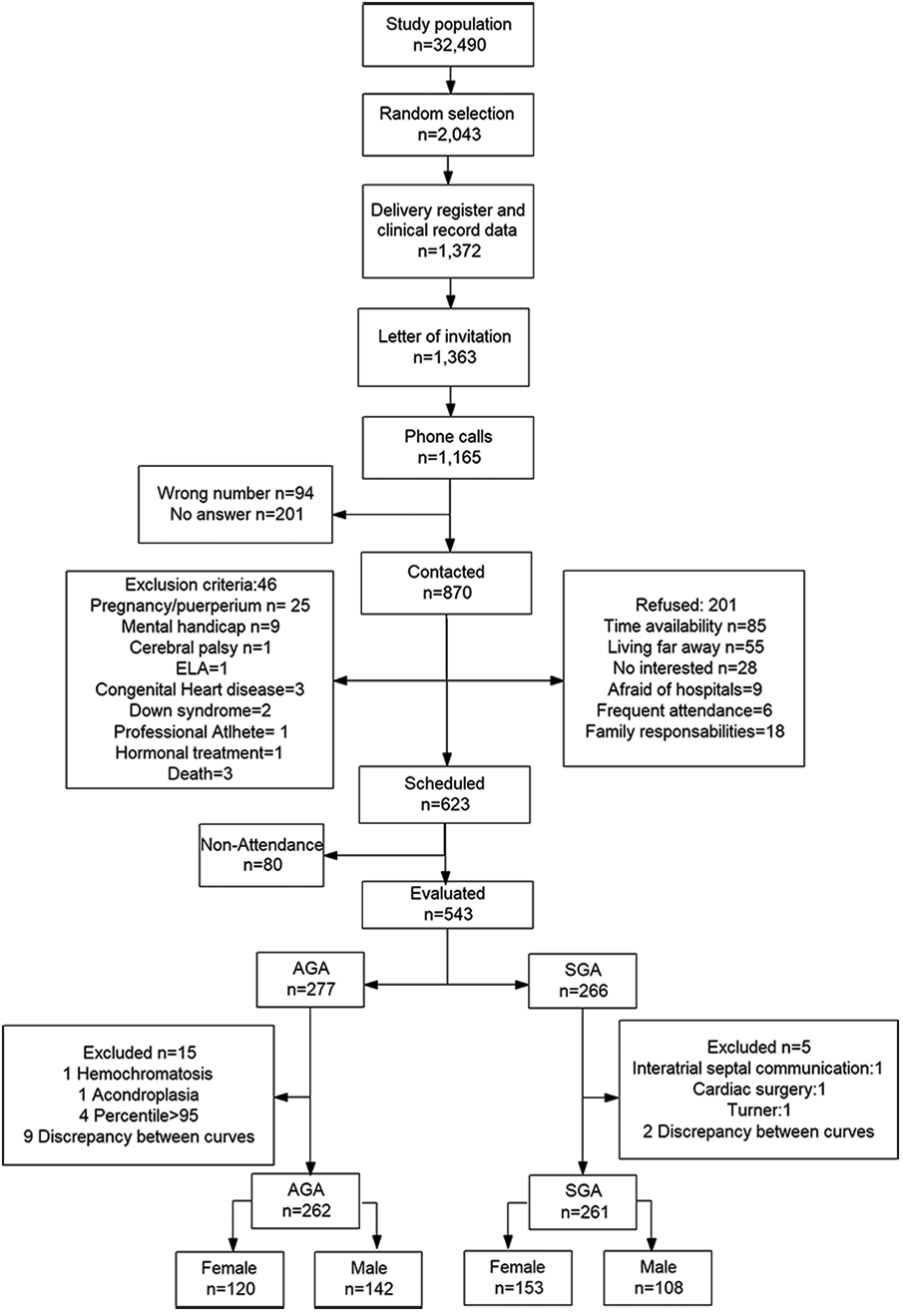
Flow diagram of the study population.

The flow diagram of the study population is shown in Figure 1. In total, 262 AGA and 261 SGA individuals were included in the analyses. The proportions of females were 58.62% and 45.80% among the SGA and AGA groups, respectively (*P* = 0.003). In all participants, biological sex assigned at birth coincided with self-reported gender at the time of the assessment. By definition, birthweight and birthweight centile were lower in the SGA group. The prevalence of preeclampsia during pregnancy and asthma was higher among those SGA. Higher prevalence of asthma was observed among the SGA group when compared to the AGA group (Table 1 and Supplementary Table SM1), with this difference more pronounced in males. The group balance obtained using IPW was adequate in both overall and stratified analyses (Supplementary Table SM2). The overall differences between the SGA and AGA groups regarding cardiovascular endpoints are shown in the supplemental material (Supplementary Tables SM3–SM6) and the results of stratified analyses are described below.

**Table 1:** Perinatal and current characteristics of the study population according to birthweight and stratified by sex.

Variable	Female		Male	
	AGA	SGA	AGA	SGA
*n*	120	153	142	108
Perinatal characteristics				
Birthweight (g)	3,377 (3,170–3,550)	2,600 (2,420–2,700)*	3,385 (3,170–3,550)	2,550 (2,350–2,680)*
Gestational age at delivery (weeks)	40 (40–41)	40 (39–41)	40 (39–41)	40 (39–41)
Birthweight centile	48 (33–70)	2 (1–3)*	46 (30.5–63)	1 (0–2)*
Preeclampsia or gestational hypertension (%)	4 (3.33)	9 (5.88)	4 (2.82)	9 (8.33)
Current characteristics				
Age (years)	30.44 (26.91–34.71)	29.03 (25.31–34.15)	32.04 (27.46–36.05)	31.54 (25.86–34.95)
Caucasian ethnicity (%)	120 (100)	151 (98.69)	142 (100)	107 (99.07)
Family history of cardiovascular disease (%)	80 (66.67)	95 (62.09)	74 (52.11)	69 (63.89)
Diabetes mellitus (%)	0	1 (0.65)	0	2 (1.85)
Chronic hypertension (%)	1 (0.83)	4 (2.61)	1 (0.70)	4 (3.70)
Asthma (%)	3 (2.50)	12 (7.84)	5 (3.52)	11 (10.19)*
Sedentary (%)	69 (58.47)	91 (65)	68 (51.91)	46 (46.46)
Current smoker (%)	25 (20.83)	46 (30.07)	46 (32.39)	42 (38.89)
Obesity (%)	12 (10)	21 (13.73)	15 (10.56)	11 (10.19)

Data are *n* (percentage), mean ± SD or median (interquartile range). AGA, appropriate fetal growth for gestational age; SGA, small for gestational age. Obesity defined as body mass index >30 Kg/m^2^.

* *P*-value <0.05 compared to AGA.

### 3.2. Cardiovascular assessment

After indexing for BSA, most LV and RV dimensions were significantly increased in SGA individuals, particularly among females (Table 2). Ventricular sphericity and relative wall thickness were similar among groups. Female born SGA showed a significant reduction in ejection fraction, compensated by increased heart rate and with preserved cardiac output. Cardiac function in male born SGA was mainly preserved except for reduced mitral displacement compared to those born with AGA. A sensitivity analysis excluding the eight female of the study group (seven from the SGA group and one from the AGA group) with reported preeclampsia during pregnancy of their own offspring showed the same direction and significance of the previously reported associations (data not shown).

**Table 2:** Sex similarities and differences in the effect of birthweight on echocardiography.

Variable	Female				Male			
	AGA	SGA	Absolute difference (95% CI)	*P* value adj.*	AGA	SGA	Absolute difference (95% CI)	*P* value adj.*
*n*	120	153			142	108		
Left morphometry								
LV basal diameter (mm/m^2^)^a^	25.46 ± 2.27	26.17 ± 2.36	**0.66** **(0.13–1.2)**	**0** **.** **015**	24.26 ± 2.09	25.18 ± 2.12	**0.66** **(0.14–1.17)**	**0** **.** **012**
LV base-to-apex length (mm/m^2^)^a^	46.88 ± 4.2	48.3 ± 5.04	**1.62** **(0.64–2.60)**	**0** **.** **001**	44.27 ± 4.28	46.03 ± 4.19	0.76 (−0.21 to 1.72)	0.124
LV sphericity index	1.84 (1.72–1.95)	1.84 (1.7–2)	0.02 (−0.03 to 0.07)	0.485	1.8 (1.7–1.94)	1.85 (1.72–1.96)	−0.02 (−0.06 to 0.02)	0.350
Relative wall thickness	0.29 (0.26–0.32)	0.29 (0.26–0.32)	−0.01 (−0.02 to 0.01)	0.368	0.31 (0.28–0.35)	0.30 (0.28–0.34)	0 (−0.02 to 0.02)	0.947
LA area (mm^2^/m^2^)^a^	9.19 (8.2–9.93)	9.23 (7.97–10.36)	0.04 (−0.39 to 0.47)	0.853	8.73 (7.45–9.77)	8.75 (7.66–9.79)	0.11 (−0.32 to 0.54)	0.608
Right morphometry								
RV basal diameter (mm/m^2^)^a^	19.94 (18.41–22.12)	20.36 (18.57–22.88)	0.70 (−0.04 to 1.45)	0.064	19.56 (18.04–21.32)	19.97 (18–21.42)	−0.36 (−1.07 to 0.35)	0.317
RV base-to-apex length (mm/m^2^)^a^	39.16 ± 4.1	40.02 ± 4.95	**1.18** **(0.19–2.16)**	**0** **.** **019**	37.06 ± 4.37	38.52 ± 4.04	0.6 (−0.46 to 1.66)	0.267
RV sphericity index	1.9 (1.77–2.15)	1.92 (1.75–2.12)	−0.01 (−0.08 to 0.06)	0.750	1.86 (1.73–2.09)	1.95 (1.77–2.11)	0.06 (0–0.13)	0.067
RA area (mm^2^/m^2^)^a^	7.03 (6.39–7.87)	7.05 (6.09–7.76)	−0.07 (−0.36 to 0.23)	0.657	7.69 (6.58–8.86)	7.17 (6.48–8.2)	−0.39 (−0.79 to 0.01)	0.057
LV function								
Ejection fraction (%)	64.41 ± 5.94	62.79 ± 6.22	**−1.73** **(−3.20 to −0.28)**	**0** **.** **020**	62.52 ± 5.79	61.43 ± 6.11	−1.33 (−3.19 to 0.52)	0.159
Heart rate (bpm)	68.55 ± 11.12	71.98 ± 11.14	**3.04** **(0.29–5.8)**	**0** **.** **031**	66.26 ± 10.76	68.60 ± 12.24	2.25 (−0.82 to 5.31)	0.150
LV cardiac output (ml/min/m^2^)^a^	2.66 (2.25–3.01)	2.65 (2.31–3.12)	0.07 (−0.07 to 0.22)	0.334	2.64 (2.28–3.09)	2.84 (2.34–3.28)	0.15 (−0.02 to 0.31)	0.089
Mitral annular plane systolic excursion (mm)	17.76 (15.74–19.09)	16.91 (15.35–18.47)	**−0.69** **(−1.28 to −0.10)**	**0** **.** **022**	17.57 (15.99–18.95)	16.5 (14.96–18.3)	**−0.73** **(−1.41 to −0.05)**	**0** **.** **036**
Mitral lateral annular peak systolic velocity (cm/s)	11 (10–12)	11 (9–12)	**−0.46** **(−0.91 to −0.02)**	**0** **.** **039**	11 (9–12)	11 (9–13)	−0.07 (−0.62 to 0.49)	0.816
LV global longitudinal strain (%)	−19.9 (−21.2 to −18.1)	−19.9 (−20.9 to −18)	−0.55 (−1.30 to 0.21)	0.155	−17.65 (−19.3 to −16.1)	−17.6 (−19.1 to −16.4)	0.06 (−0.64 to 0.75)	0.882
Mitral E/A	1.63 (1.36–1.84)	1.65 (1.38–2.02)	0.09 (−0.02 to 0.2)	0.092	1.53 (1.29–1.81)	1.6 (1.3–1.87)	−0.04 (−0.14 to 0.06)	0.452
Isovolumic relaxation time (ms)	66 (56–78)	65 (56–79)	0.16 (−3.67 to 3.98)	0.937	69 (59–81)	69 (60–85)	1.35 (−3.21 to 5.92)	0.562
RV function								
Fractional area change (%)	45.70 ± 8.05	47.70 ± 8.67	1.84 (−0.26 to 3.92)	0.085	43.46 ± 8.42	43.13 ± 7.96	−0.84 (−3.00 to 1.32)	0.448
Tricuspid annular plane systolic excursion (mm)	24.82 ± 3.32	24.41 ± 3.34	−0.46 (−1.3 to 0.38)	0.287	25.24 ± 3.43	24.4 ± 3.59	−0.85 (−1.79 to 0.1)	0.078
Tricuspid annular peak systolic velocity (cm/s)	13 (12–15)	13 (11.5–14.5)	**−0.78** **(−1.28 to −0.28)**	**0** **.** **002**	14 (13–15)	13 (12–15)	0.02 (−0.53 to 0.56)	0.956
Tricuspid E/A	1.75 (1.53–2.04)	1.66 (1.37–2.07)	−0.06 (−0.18 to 0.06)	0.358	1.66 (1.36–1.84)	1.62 (1.33–2.07)	0.01 (−0.12 to 0.14)	0.878

Bold indicate statistically significant results.

AGA, appropriate fetal growth for gestational age; SGA, small for gestational age; LV, left ventricle; LA, left atria; RV, right ventricle; RA, right atria; E, early diastole; A, atrial contraction. Data are mean ± SD or median (interquartile range).

* *P*-value adj. calculated using propensity score model included family cardiovascular history, gestational hypertension including preeclampsia, current age, overweight/obesity, chronic hypertension, asthma, smoking habit, and physical activity.

^a^ Cardiac dimensions were indexed by body surface area.

**Figure 2:**
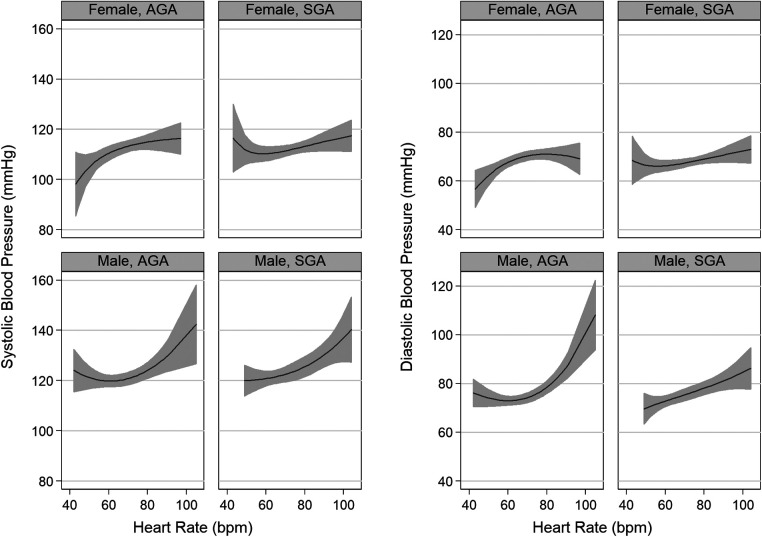
Relationship between heart rate and blood pressure by categories of sex and small for gestational age status.

A positive correlation between blood pressure and heart rate was observed if heart rate values were above 60 bpm (Figure 2). Regarding the ECG results, a reduction in the duration of the P and QRS waves and PR interval were observed in the SGA group, with more pronounced changes in males (Table 3). There was a non-significant trend to higher values of blood pressure in male born SGA compared to those born with AGA (Table 4). We found no differences between males and females regarding the effect of SGA on cIMT.

**Table 3:** Sex similarities and differences in the effect of birthweight on electrocardiogram.

Variable	Female				Male			
	AGA	SGA	Absolute difference (95% CI)	*P* value adj.*	AGA	SGA	Absolute difference (95% CI)	*P* value adj.*
*n*	120	153			142	108		
P wave (ms)	100 (90–104)	98 (90–100)	**−2.13** **(−4.06 to −0.19)**	**0** **.** **032**	102 (98–110)	100 (96–104)	**−3.17** **(−5.5 to −0.83)**	**0** **.** **008**
PR interval (ms)	140 (128–150)	134 (122–148)	−1.36 (−6.15 to 3.42)	0.576	142 (132–160)	138 (128–148)	**−6.61** **(−11.67 to −1.54)**	**0** **.** **011**
QRS wave (ms)	90 (86–96)	88 (84–94)	−1.39 (−3.43 to 0.65)	0.181	100 (94–106)	98 (92–104)	**−2.33** **(−4.65 to −0.01)**	**0** **.** **049**
QTC interval (ms)	416 (406–434)	419.5 (407–431.5)	0.89 (−3.57 to 5.35)	0.696	409 (397–423)	409.5 (396.5–423)	2.40 (−3.57 to 8.37)	0.430

Bold indicate statistically significant results.

Data are median (interquartile range). AGA, appropriate fetal growth for gestational age; SGA, small for gestational age.

* *P*-value adj. calculated using propensity score model included family cardiovascular history, gestational hypertension including preeclampsia, current age, overweight/obesity, chronic hypertension, asthma, smoking habit, and physical activity.

**Table 4:** Sex similarities and differences in the effect of birthweight on blood pressure and vascular structure and function by ultrasound.

Variable	Female				Male			
	AGA	SGA	Absolute difference (95% CI)	*P* value adj.*	AGA	SGA	Absolute difference (95% CI)	*P* value adj.*
*n*	120	153			142	108		
Blood pressure								
Systolic blood pressure (mmHg)	111.67 (106–119.67)	112.67 (105.83–119.67)	0.06 (−2.7 to 2.81)	0.967	121.33 (113.67–128.67)	123 (114.33–130.33)	2.71 (−0.48 to 5.9)	0.096
Diastolic blood pressure (mmHg)	69.33 (62–75)	68 (61.67–72.83)	−0.85 (−2.96 to 1.26)	0.432	75 (68.67–80)	74 (69.67–80.67)	1.17 (−1.36 to 3.69)	0.365
Vascular ultrasound								
Carotid intima-media thickness (mm)	0.48 (0.45–0.53)	0.47 (0.44–0.50)	−0.0 (−0.02 to 0.01)	0.223	0.51 (0.47–0.55)	0.50 (0.46–0.56)	0.01 (−0.01 to 0.03)	0.446

Data are median (interquartile range). AGA, appropriate fetal growth for gestational age; SGA, small for gestational age.

* *P*-value adj. calculated using propensity score model included family cardiovascular history, gestational hypertension including preeclampsia, current age, overweight/obesity, chronic hypertension, asthma, smoking habit, and physical activity.

### 3.3. Anthropometric and metabolic assessment

As expected, SGA individuals had lower height and weight (Table 5). While body mass index was similar among the study populations, both females and males born SGA showed a significant increase in waist-to-hip ratio. SGA males also had higher central-to-peripheral fat ratios and fat mass percentages. Laboratory results on glycemia and lipids profile were similar among the study groups; a slight increment in triglyceride values was observed in SGA males.

**Table 5:** Sex similarities and differences in the effect of birthweight on anthropometric and metabolic assessment.

Variable	Female				Male			
	AGA	SGA	Absolute difference (95% CI)	*P* value adj.*	AGA	SGA	Absolute difference (95% CI)	*P* value adj.*
*n*	120	153			142	108		
Current anthropometry								
Height (m^2^)	165 (161–169.5)	158.4 (155.5–162.8)*	**−5.60** **(−7.10 to −4.14)**	**<0** **.** **001**	177 (172.5–181.5)	171.3 (167.4–176.25)*	**−5** **(−6.60 to −3.40)**	**<0** **.** **001**
Weight (kg)	60.45 (55.15–68.2)	54.9 (49.4–65.6)*	**−4.05** **(−7.39 to −0.70)**	**0** **.** **018**	78.95 (72.3–87.2)	72.15 (62.9–81.6)*	**−5.94** **(−9.37 to −2.51)**	**0** **.** **001**
Body mass index (kg/m^2^)	22.13 (20.25–24.85)	21.51 (19.69–25.41)	0.06 (−0.71 to 0.82)	0.888	25.26 (23.01–27.62)	24.21 (22.01–26.38)	0.43 (−0.36 to 1.23)	0.289
Waist circumference (cm)	73 (68.4–79)	71.8 (67–80)	−0.13 (−1.86 to 1.59)	0.878	86.65 (81–93)	85 (78–91)	1.02 (−1.27 to 3.31)	0.383
Waist-to-hip ratio	0.73 (0.71–0.77)	0.75 (0.72–0.79)	**0.02** **(0.01–0.03)**	**<0** **.** **001**	0.85 (0.82–0.89)	0.86 (0.83–0.90)	**0.02** **(0.010–0.04)**	**0** **.** **038**
Central-to-peripheral ratio	1.13 (0.95–1.39)	1.18 (0.98–1.51)	0.05 (−0.03 to 0.12)	0.243	1.98 (1.63–2.51)	2.17 (1.72–2.96)	**0.40** **(0.17–0.62)**	**<0** **.** **001**
Fat mass (%)	26.3 (21.16–32.7)	23.7 (18.5–31.8)	−1.47 (−3.08 to 0.14)	0.073	18.5 (14.8–23.7)	18.1 (14–22.4)	**1.32** **(0.07–2.56)**	**0** **.** **038**
Fat mass (Kg)	16.1 (12.1–22.8)	13.4 (8.9–21.1)*	−2.04 (−3.7 to −0.39)	0.015	14.95 (11.15–20.5)	13.1 (9–17.2)	0.28 (−1.47 to 2.04)	0.749
Lean mass (Kg)	44.55 (42.75–46.95)	42.4 (40.3–45.2)	−2.25 (−3.11 to −1.38)	**<0** **.** **001**	64.9 (60.2–68.1)	58.1 (54.05–63.25)	−4.20 (−5.84 to −2.56)	**<0** **.** **001**
Muscular mass (Kg)	42.25 (40.55–44.55)	40.2 (38.2–42.9)	−2.14 (−2.97 to −1.32)	**<0** **.** **001**	61.7 (57.25–64.7)	55.3 (51.7–60.1)	−3.58 (−5 to −2.17)	**<0** **.** **001**
Peripheral blood biomarkers								
Glucose (mg/dl)	89 (82–96)	87 (80–94)	−2.68 (−5.7 to 0.33)	0.081	88 (82–96)	90 (85–97.5)	−0.05 (−3.39 to 3.28)	0.975
Cholesterol HDL (mg/dl)	54 (45–63)	52 (46–62)	−1.26 (−4.68 to 2.14)	0.466	30 (34–47)	40.5 (25–47)	0.33 (−2.67 to 3.33)	0.830
Cholesterol LDL (mg/dl)	108.9 (98.8–128.8)	110.5 (94.1–126.4)	−1.02 (−7.58 to 5.52)	0.759	111.2 (92.8–132.8)	114.2 (95.2–135)	4.55 (−4.75 to 13.86)	0.338
Triglycerides (mg/dl)	85 (62–106)	82 (62–103)	0 (−0.10 to 0.10)^a^	0.976	104 (74–158)	108 (79–154)	0.15 (0.02–0.28)^a^	**0** **.** **020**

Bold indicate statistically significant results.

Data are median (interquartile range). AGA, appropriate fetal growth for gestational age; SGA, small for gestational age.

* *P*-value adj. calculated using propensity score model included family cardiovascular history, gestational hypertension including preeclampsia, current age, overweight/obesity, chronic hypertension, asthma, smoking habit, and physical activity.

^a^ Dependent variable was log-transformed for IPW analysis.

## 4. Discussion

Our study first describes sex differences in the effect of SGA on adult cardiometabolic health. Females born SGA were more susceptible to cardiac dysfunction, while SGA males were more prone to changes in electrocardiogram, blood pressure, and metabolic markers. These results draw attention to the important role of intrauterine conditions in understanding the sex differences in CVDs in adulthood.

### 4.1. Signs of cardiac dysfunction was mainly observed in females

Our echocardiographic data is in line with previous reports suggesting subtle cardiac structural and functional changes in adults related to fetal growth (13, 33). Both males and females born SGA showed subtle increases in indexed ventricular dimensions, with changes being more pronounced in female. In addition, we first identified SGA female to be more susceptible to cardiac dysfunction. Ejection fraction was preserved (>40%) in all participants; however, females born SGA showed a reduction in ejection fraction compensated by a heart rate increase. These changes were less pronounced and not statistically significant in males. While systolic dysfunction has been described to be more associated with male sex (34), a previous study in older patients with preserved ejection fraction showed more frequent diastolic dysfunction and readmission for heart failure in female than male (35). These findings are also consistent with female being more prone to heart failure (5).

There is controversy regarding the sex differences in the pattern of cardiac remodeling. For example, preclinical echocardiographic changes are mainly characterized by a dilated left atrium in female and by left ventricular hypertrophy in male (36); however, it has also been suggested that hypertrophy is more common and less modifiable in female (37). This discordance could be explained by differences with the study population, as in most published reports female are aged >40 years. Likewise, an animal model of hypoxia demonstrated that males had smaller body weights and signs of left ventricular hypertrophy, but both males and females showed signs of left ventricular diastolic dysfunction by 12 months of age (21). Animal models of fetal programming (38–40) have suggested a protective role of estrogens in females exposed to fetal undernutrition, which was not the case for some cardiac functional parameters in our study.

In addition to previous knowledge, SGA female presented the highest heart rate at rest, which is an independent risk factor for CVD (41). A similar response was reported for occupational stress, where female individuals have a larger percent change in heart rate compared with males (42). These results are in line with previous findings suggesting lower baseline heart rates in male from birth onward (43), even after stratification by race (44). Our sensitivity analysis suggested that pregnancy-related disorders (45) did not account for the observed sex differences in systolic function, nor were the differences explained by the severity of the restriction, as the differences in birthweight and gestational age between SGA and AGA were similar in male and female.

### 4.2. Electrocardiogram changes were predominant in males

We previously described the presence of electrical remodeling in SGA and preterm preadolescents (46) overall. The present study in adulthood further identifies male born SGA being more susceptible to short PR and QRS intervals. The absolute difference of −6.61 in male indicates that, on average, being SGA leads to a decrease in PR interval of about 7 ms. Our results are in line with previous literature reporting more prevalent ventricular pre-excitation in male than in female (47). However, the pattern seems to be different, as QRS interval was decreased in SGA population (48). It has also been reported that female have smaller calcium currents and lower excitation-contraction coupling gain than male (49).

Despite prolonged PR intervals being a risk factor for atrial fibrillation incidence (50), a U-shaped relationship has been found with an increased risk for individuals with short PR intervals (51), which was the case of our study. In addition, a non-significant increment in QTc, a potential risk factor for ventricular tachycardia and sudden cardiac death (52), was observed among SGA male. Overall, these findings suggest a greater impact of SGA on electrical remodeling among SGA male compared with SGA females.

### 4.3. Higher blood pressure was observed in males

Even though males have higher blood pressure than age-matched females during early adulthood (53), our study showed those that who were born SGA presented the highest systolic values. While statistically non-significant, the average systolic blood pressure in male SGA was 3 mmHg higher than AGA, with a mean absolute difference of 2.71. This difference seems to be clinically relevant considering that every 2 mmHg increases the risk of fatal stroke and fatal coronary heart disease by 7% and 5%, respectively (54). Human data on hypertension from prenatal origins are controversial. Similar differences in the effect of a birth condition have been reported among adult preterm males (55) and females (56). An increased risk of systolic hypertension without any effect on diastolic blood pressure has been also reported among preterm individuals (57), with an increase in both systolic and diastolic blood pressure in the general adolescent population (58).

Overall, our results reinforce previous findings suggesting that smaller size at birth is associated with higher arterial pressure in males and an increased cardiac sympathetic activation among females (59). Heart rate and blood pressure are both important hemodynamic parameters which are positive correlated with each other in children and adults (60). Our findings suggest that the effect of sex on this correlation seems to be higher than the effect of SGA. However, the direction and strength of the correlation between both parameters might vary whether the heart rate frequency is above or below 60 bpm. Future studies are needed to validate this observation.

These results are consistent with previous papers also suggesting no differences in intima-media thickness in adults born SGA (61). These findings conflict prenatal and pediatric data consistently demonstrating an increased vascular IMT in fetuses, neonates, and children born SGA. This inconsistency may be explained by differences in the study populations (less severe phenotypes in adult cohorts) and the relatively stronger influence of adult lifestyle on IMT.

### 4.4. Metabolic changes were more pronounced in males

Last but not least, our adjusted estimates showed a greater reduction in lean mass, an increased fat mass percentage, and altered fat distribution together with higher central adiposity in SGA male. These results are in line with previous reports on preterm males (62, 63). It has been demonstrated that birth size and adult adiposity independently predict events of cardiovascular disease after controlling for potential confounders (64); therefore, both exposures, prematurity and SGA, could play a crucial role in the pathogenesis of CVD in male. Male sex is an independent risk factor for all components of the metabolic syndrome except for low HDL in subjects with a normal body mass index (65) and our study shows a trend toward a greater lipid profile risk among SGA male. Our results are in line with a previous study reporting young female had a higher prevalence of obesity and low High-density lipoprotein cholesterol (HDL-C), while younger male had a higher prevalence of elevated blood pressure and elevated triglycerides (66). Our study additionally supports a sex-specific effect of SGA on cardiovascular health.

### 4.5. Strengths and limitations

The effect of fetal programming in humans has been mainly apprised by traditional risk factors and vascular parameters (14, 15, 39, 67). To our knowledge, this is the first study showing a sex difference on the effect of fetal programming related to SGA and including a wider variety of risk factors in young adulthood (68). We followed current recommendation regarding the need of reporting sex-disaggregated results even when the chance of finding a sex difference is small (69). In our study, females were not under-represented as in most of the heart failure studies (70). In addition, all participants underwent all cardiometabolic tests, overcoming potential sex biases such as patient or clinician awareness.

However, there are some limitations to be acknowledged. Firstly, sex stratification was not considered in the sample size calculation, and therefore, the results should be considered as exploratory. Secondly, measurement bias could be present for some covariates that were self-reported, including physical activity and smoking. SGA can be considered as a “proxy” of fetal growth restriction, as this definition was exclusively percentile-based; at the time the participants were born, the fetoplacental ultrasound-based definition was not available. Thirdly, participants and those who declined to participate were similar regarding birthweight gestational age and birthweight percentile; however, we could not rule out selection bias due to other postnatal risk factors. Fourthly, despite no female reported systemic autoimmune diseases and only one AGA female reported polycystic ovary syndrome, a causal relationship also requires assumptions about the absence of other confounders across the life course like dietary intake, air pollution exposure, psychological stress, childhood obesity, or early menarche, which we could not control for. Currently, more extreme SGA cases might have greater likelihood of survival; this might explain at least in part the greater difference between SGA and AGA of more recent cohorts of birth (12, 24, 71). Finally, the observed changes are within the normal range of the studied parameters.

### 4.6. Potential mechanisms and clinical relevance of sex stratification

The underlying mechanisms of sex differences in CVD are still incompletely understood. Potential explanations include the role of risk factors limited to or more frequent in females: pregnancy, preeclampsia, iron deficiency, contraception, hormone replacement therapy, or autoimmune disorders (8). Molecular mechanisms include an activated renin-angiotensin-aldosterone system in response to low estrogen during menopause, the downregulation by estrogen of proteins related to diastolic function, the progesterone activation of the extracellular signal-regulated kinase 2 inducing cardiac hypertrophy during pregnancy, and lower excitation-contraction mediated by calcium and glucose uptake and utilization compared with male (72). An atheroprotective effect of estrogen in young female (73), and the effect of sex steroids on blood pressure-controlling mechanisms have been also postulated (74). Whether these mechanisms also differ by SGA status or whether prenatal epigenetic changes and the reduction in gonadal organ size associated with SGA contribute differently to sex differences in fetal cardiovascular programming need to be elucidated.

There is a need to better study women's hearts. Females are underrepresented across all aspects of cardiovascular research; therefore, they are more likely to be misdiagnosed (37) or to receive less optimized medical treatment (75). Additionally, common risk factors such as tobacco and type-2 diabetes are more harmful to the cardiovascular system of female (76). More recently, female were at increased risk of acute heart failure and in-hospital mortality due to COVID-19 compared with male (77). Taken together, our results reinforce the need for equitable access to cardiovascular prevention services across the life course, and to improve inclusion, accuracy, and reproducibility of women's cardiovascular research. Further studies explore the role of SGA on the higher prevalence of heart failure with preserved ejection fraction among female and sudden cardiac death among male. Last but not least, we observed a higher prevalence of asthma in SGA males, which might require further research.

## 5. Conclusion

In conclusion, SGA could affect the cardiometabolic health in adulthood through differential and sex-specific physio-pathological pathways. SGA can contribute to different patterns of CVD observed among males and females. While cardiovascular structural and systolic functional remodeling was more relevant among females, changes in PR interval, central adiposity, and a blood pressure was more relevant among males. We encourage the need for a sex-specific approach in cardiovascular medicine (78) even beyond treatment. Future studies are warranted to validate our results and to stablish the need to include SGA backgrounds for sex-specific diagnostic criteria and therapies.

## Data Availability

The raw data supporting the conclusions of this article will be made available by the authors, without undue reservation.
