# Supplementary material for: Cardiometabolic sex differences in adults born small for gestational age
Source: Front Cardiovasc Med. 2023 Oct 25;10:1223928. doi: 10.3389/fcvm.2023.1223928 (PMC10634502; doi:10.3389/fcvm.2023.1223928)
Supplement: Supplementary file 1 [file Datasheet1.docx]

Supplementary Material

# Cardiometabolic sex differences in adults born small for gestational age

Mérida Rodríguez-López*. Álvaro Sepúlveda-Martínez. Gabriel Bernardino. Francesca Crovetto. Carolina Pajuelo. Marta Sitges. Bart Bijnens. Eduard Gratacós . Fàtima Crispi*

**Supplementary Figures and Tables**

**Table SM1.** Perinatal and current baseline characteristics of the study population according to small for gestational age and sex categories.

|  | **Adequate for gestational age (AGA)**  **N=262** | **Small for gestational age (SGA)**  **N=261** | **Female**  **N=273** | **Male**  **N=250** |
| --- | --- | --- | --- | --- |
| **Perinatal characteristics** | | | | |
| Birthweight (g) | 3377 (3170-3550) | 2600 (2400-2700)* | 2750(2550-3300) | 3050(2570-3400) |
| Gestational age at delivery (weeks) | 40 (39-41) | 40 (39-41) | 40 (39-41) | 40 (39-41)*^┼^ |
| Preeclampsia or hypertension in pregnancy | 8 (3.05) | 18 (6.90)* | 13(4.76) | 13 (5.20) |
| Family history of CVD | 154(58.78) | 164(62.84) | 175(64.10) | 143(57.20) |
| **Current characteristics** | | | | |
| Age (years) | 31.25(27.15-35.38) | 29.71 (25.63-34.54) | 29.78(25.78-34.55) | 31.95(26.24-35.54) |
| Age ≥30 years | 156(59.54) | 125(47.89)* | 131(47.99) | 150 (60)* |
| Male | 142(54.20) | 108(41.38)* | NA | NA |
| Caucasian ethnicity | 262 (100) | 258(98.85) | 271(99.27) | 249(99.60) |
| Height (m^2^) | 1.72 (1.65-1.78) | 1.64 (1.58-1.70)* | 161(157-166) | 174.5 (170.2-179)* |
| Weight (kg) | 72.05 (60.90-81.90) | 62.5 (53-74.4)* | 58.5(51.7-67) | 76(68.7-84.7)* |
| Body surface area (m^2^) | 1.84 (1.67-1.98) | 1.69 (1.54-1.86)* | 1.62(1.52-1.73) | 1.92(1.82-2.02)* |
| Obesity or overweight | 106(40.46) | 82 (31.42) * | 70(25.64) | 118(47.20)* |
| Less active or inactive physical activity | 137(55.02) | 137(57.32) | 160(62.02) | 114(49.57)* |
| Current smoker | 71 (27.10) | 88 (33.42) | 71(26.01) | 88(35.20)* |
| Diabetes mellitus | 0 (0) | 3 (1.15) | 1(0.37) | 2(0.80) |
| Chronic hypertension | 2(0.76) | 8 (3.07) | 5(1.83) | 5(2.00) |
| Asthma | 8(3.05) | 23(8.81)* | 15(5.49) | 16(6.40) |

Data are median (interquartile range) or n (percentage). NA denotes non-applicable.

*P<0.05 SGA vs AGA or male vs female ^┼^ Medians are equal but spreads of distributions are different.(1)

**Table SM2** Weighted Standardized mean differences (SMD) and variance ratio for the variables used to obtain the Propensity Score for cardiovascular remodeling.

| **Variable** | **Overall** | | **Female** | | **Male** | |
| --- | --- | --- | --- | --- | --- | --- |
|  | **SMD** | **Variance** | **SMD** | **Variance** | **SMD** | **Variance** |
| Sex | 0.008 | 1.000 | - |  | - |  |
| Age ≥30 years | -0.005 | 1.000 | -0.013 | 0.999 | 0.019 | 0.990 |
| Preeclampsia or hypertension in pregnancy | -0.022 | 0.911 | -0.003 | 0.988 | 0.015 | 1.067 |
| Family history of CVD | -0.009 | 1.003 | 0.003 | 0.998 | -0.013 | 1.003 |
| Current smoker | -0.003 | 0.997 | -0.005 | 0.994 | -0.015 | 0.989 |
| Obesity or overweight | 0.012 | 1.007 | 0.006 | 1.008 | 0.024 | 1.000 |
| Less active or inactive physical activity | -0.002 | 1.000 | 0.006 | 0.996 | -0.009 | 0.999 |
| Chronic hypertension | -0.018 | 0.864 | -0.003 | 0.972 | 0.041 | 1.424 |
| Asthma | 0.014 | 1.060 | 0.039 | 1.200 | 0.003 | 1.013 |
| Overidentification test (p-value) | 0.602 | | 0.798 | | 0.663 | |

**Table SM3.** Effect of small for gestational age status on cardiovascular structure and function.

| **Variable** | **AGA (n=262)** | **SGA (n=261)** | **Pvalue** | **Absolute difference (95%CI)** | **Pvalue Adj** |
| --- | --- | --- | --- | --- | --- |
| **Left morphometry** | | | | | |
| LV basal diameter (mm/m^2^) | 24.81(2.25) | 25.76(2.31) | 0.000 | 0.67(0.30-1.05) | **0.000** |
| LV base-to-apex lenght (mm/m^2^) | 45.46(4.43) | 47.35(4.83) | 0.000 | 1.19(0.51-1.87) | **0.001** |
| LV sphericity index | 1.82(1.71-1.95) | 1.85(1.7-1.97) | 0.610 | 0(-0.03-0.03) | 0.928 |
| Relative wall thickness | 0.3(0.27-0.34) | 0.3(0.26-0.33) | 0.173 | 0(-0.01-0.01) | 0.632 |
| LA area (mm^2^/m^2^) | 8.95(7.74-9.89) | 9.01(7.93-10.08) | 0.347 | 0.09(-0.21-0.39) | 0.561 |
| **Right morphometry** | | | | | |
| RV basal diameter (mm/m^2^) | 19.82(18.32-21.77) | 20.31(18.2-22.22) | 0.196 | 0.20(-0.31-0.7) | 0.450 |
| RV base-to-apex lenght (mm/m^2^) | 38.04(4.37) | 39.4(4.65) | 0.001 | 0.88(0.18-1.58) | **0.014** |
| RV sphericity index | 1.88(1.74-2.11) | 1.93(1.76-2.11) | 0.179 | 0.02(-0.03-0.07) | 0.362 |
| RA area (cm^2^/m^2^) | 7.35(6.41-8.34) | 7.09(6.21-8.02) | 0.027 | -0.20(-0.44-0.04) | 0.104 |
| **LV Function** | | | | | |
| Ejection fraction (%) | 63.38(5.92) | 62.22(6.2) | 0.030 | -1.52(-2.64--0.39) | **0.008** |
| Heart rate (bpm) | 67.31(10.97) | 70.58(11.71) | 0.001 | 2.63(0.64-4.62) | **0.010** |
| LV cardiac output (mL/min/m^2^) | 4.87(4.08-5.66) | -0.27(-0.61-0.08) | 0.133 | -0.02(-0.23-0.18) | 0.816 |
| Mitral annular plane systolic excursion (mm) | 17.52(2.42) | 16.79(2.53) | 0.001 | -0.69(-1.15--0.24) | **0.003** |
| Mitral lateral annular peak systolic velocity (cm/s) | 11(10-12) | 11(9-12) | 0.105 | -0.26(-.061 to -0.02) | **0.039** |
| LV global longitudinal strain (%) | -18.8(-20.4--16.7) | -18.9(-20.5--17.2) | 0.448 | -0.18(-0.65 to 0.30) | 0.471 |
| Mitral E/A | 1.58(1.32-1.83) | 1.62(1.34-1.95) | 0.183 | 0.03(-0.05-0.1) | 0.487 |
| Isovolumetric relaxation time (ms) | 68.5(57.5-79) | 66(57-83) | 0.924 | 0.46(-2.47 to 3.39) | 0.757 |
| **RV function** | | | | | |
| Fractional area change (%) | 44.51(8.31 | 45.84(8.67) | 0.079 | 0.83 (-0.06-2.35) | 0.279 |
| Tricuspid annular plane systolic excursion (mm) | 25.04(3.38) | 24.41(3.44) | 0.033 | -0.65(-1.29--0.02) | **0.045** |
| Tricuspid annular peak systolic velocity (cm/s) | 14(12-15) | 13(12-15) | 0.005 | -0.39(-0.77--0.02) | **0.040** |
| Tricuspid E/A | 1.68(1.45-2) | 1.64(1.36-2.07) | 0.522 | -0.02(-0.11-0.06) | 0.602 |

Data are mean±SD or median (interquartile range).

*P-value Adj calculated using propensity score model included family cardiovascular history. gestational hypertension including preeclampsia. current age. sex. overweight/obesity. chronic hypertension. asthma. smoking habit and physical activity.

LV indicates left ventricle; RV. right ventricle; CO. cardiac output. E. early diastole; A. atrial contraction

Ejection fraction estimated by 2D biplane Simpson.

**Table SM4.** Effect of small for gestational age status on electrocardiogram

| **Variable** | **AGA (n=262)** | **SGA (n=261)** | **Pvalue** | **Absolute difference (95%CI)** | **Pvalue Adj** |
| --- | --- | --- | --- | --- | --- |
| P wave (ms) | 100(94-108) | 100(92-102) | 0.000 | -2.55(-4.13--0.98) | **0.001** |
| PR Interval (ms) | 140(130-156) | 136(124-148) | 0.001 | -3.71(-7.2--0.23) | **0.037** |
| QRS wave ms) | 96(88-102) | 92(86-98) | 0.000 | -1.72(-3.27--0.17) | **0.030** |
| QTC Interval (ms) | 412(400-426) | 416 (403-429) | 0.141 | 3.05(-0.75-6.86) | **0.116** |

Data are mean±SD or median (interquartile range).

*P-value Adj calculated using propensity score model included family cardiovascular history. gestational hypertension including preeclampsia. current age. sex. overweight/obesity. chronic hypertension. asthma. smoking habit and physical activity.

**Table SM5.** Effect of small for gestational age status on blood pressure and vascular structure and function by ultrasound.

| **Variable** | **AGA (n=262)** | **SGA (n=261)** | **Pvalue** | **Absolute difference (95%CI)** | **Pvalue Adj** |
| --- | --- | --- | --- | --- | --- |
| **Blood Pressure** | | | | | |
| Systolic blood pressure (mmHg) | 116.67(108.67-125.67) | 116.33(108.67-123.67) | 0.517 | 1.48(-0.62-3.58) | 0.167 |
| Diastolic blood pressure (mmHg) | 71.67(65.67-78.33) | 70.67(64-76.33) | 0.066 | 0.21(-1.4-1.82) | 0.801 |
| **Vascular assestment** | | | | | |
| Carotid intima-media thickness (mm) | 0.50(0.46-0.54) | 0.48(0.45-0.52) | 0.037 | -3.3*10-3(-0.02-0.01) | 0.586 |

Data are mean±SD or median (interquartile range).

*P-value Adj calculated using propensity score model included family cardiovascular history. gestational hypertension including preeclampsia. current age. sex. overweight/obesity. chronic hypertension. asthma. smoking habit and physical activity.

**Table SM6.** Effect of small for gestational age status on current anthropometry and laboratory results.

| **Variable** | **AGA (n=262)** | **SGA(n=261)** | **Pvalue** | **Absolute difference (95%CI)** | **Pvalue Adj** |
| --- | --- | --- | --- | --- | --- |
| **Current anthropometry** | | | | | |
| Body mass index (kg/m^2^) | 24.24(21.36-26.74) | 23.18(20.53-25.92) | 0.022 | 0.29(-0.28-0.87) | 0.313 |
| Waist circumference (cm) | 81(73.5-90) | 78(69.5-87.15) | 0.006 | -0.25(-1.15-1.65) | 0.727 |
| Waist-to-hip ratio | 0.80(0.73-0.86) | 0.79(0.74-0.86) | 0.968 | 0.02(0.01-0.03) | **0.001** |
| Central to peripheral ratio | 1.51(1.13-2.03) | 1.52(1.09-2.06) | 0.669 | 0.20(0.09-0.31) | **0.000** |
| Fat mass (%) | 22.05(17-27.4) | 21(16.5-28.2) | 0.393 | -0.11(-1.17-0.94) | 0.836 |
| Fat mass (Kg) | 15.6(11.5-21.6) | 13.25(8.9-19.3) | 0.002 | -0.92(-2.14-0.30) | 0.138 |
| Lean mass (kg) | 56.5(44.7-65.1) | 47.1(41.8-56.6) | 0.000 | -3.13(-4.04--2.22) | **0.000** |
| Muscular mass (Kg) | 53.7(42.45-61.95) | 44.8(39.8-54) | 0.000 | -2.79(-3.60--1.99) | **0.000** |
| **Blood biomarkers** | | | | | |
| Glucose (mg/dl) | 88.5(82-95.5) | 88(81-95) | 0.260 | -1.30(-3.56 to 0.96) | 0.260 |
| Cholesterol HDL (mg/dl) | 45(35-56.5) | 47(39-56) | 0.114 | -0.49(-2.79 to1.81) | 0.676 |
| Cholesterol LDL (mg/dl) | 110(95.2-131.4) | 111(94.4-128.8) | 0.877 | 1.87(-3.57-7.31) | 0.501 |
| Triglycerides (mg/dl) | 94.5(67-133.5) | 88(70-120) | 0.512 | 0.07(-0.02-1.15)^┼^ | 0.122 |

Data are mean±SD or median (interquartile range).

*P-value Adj calculated using propensity score model included family cardiovascular history. gestational hypertension including preeclampsia. current age. sex. overweight/obesity. chronic hypertension. asthma. smoking habit and physical activity. ^┼^Dependent variable was log-transformed for IPW analysis

References

1. Hart A. Mann-Whitney test is not just a test of medians: differences in spread can be important. BMJ. 2001;323(7309):391-3.
